# The Role of Bone Marrow-Derived Mesenchymal Stromal Cells and Hesperidin in Ameliorating Nephrotoxicity Induced by Cisplatin in Male Wistar Rats

**DOI:** 10.22088/IJMCM.BUMS.10.2.133

**Published:** 2021-09-01

**Authors:** Khalid Mohamed Mazher, Osama Mohamed Ahmed, Hadeer Abdallah Sayed, Taghreed Mohamed Nabil

**Affiliations:** 1 *Department of Cytology and Histology, Faculty of Veterinary Medicine, * *Beni-Suef * *University, 62511, Beni-Suef, Egypt.*; 2 *Physiology Division, Zoology Department, Faculty of Science, Beni-Suef University, 62521, Beni-Suef, Egypt.*; 3 *Faculty of Veterinary Medicine, Beni-Suef University, 62511, Beni-Suef, Egypt. *

**Keywords:** Bone marrow-derived mesenchymal stromal cells, cisplatin, hesperidin, immunohistochemistry, nephrotoxicity, p53, apoptosis

## Abstract

Bone marrow-derived mesenchymal stromal cells (BM-MSCs) and antioxidants opened the way for many effective therapeutic experiments against damaged organs like kidneys. Nephrotoxicity is the main complication of chemotherapeutic drugs. Therefore, the present study aimed to investigate the efficacy of BM-MSCs and hesperidin to treat cisplatin-induced nephrotoxicity in rats. Fifty rats were divided into five equal groups of 10 each. Group-I served as a control group, group-II received a single dose of cisplatin (7.5 mg/kg) intraperitoneally to induce nephrotoxicity, group-III received a daily dose of hesperidin (40 mg/kg) orally for four weeks, and on the 5^th ^day cisplatin was administered an hour before hesperidin administration. Group-IV consisted of cisplatin-treated rats that were intravenously injected with 1х10^6^ BM-MSCs cells/rat once per week. Group V contained cisplatin-treated rats that received a combination of hesperidin and BM-MSCs with the same dosage regimes. After four weeks, serum and kidney samples were collected for biochemical, histological, and immunohistochemical examinations were performed. Cisplatin administered rats showed deteriorated biochemical parameters and severe degenerative changes in renal tissue. Both single and combined hesperidin and BM-MSCs treatments restored the renal biochemical parameters. Histologically, the renal tissues significantly improved in the BM-MSCs treated group in comparison with the hesperidin treated group. Moreover, combined treatment (i.e., group V) showed complete restoration of the normal architecture in the renal tissue. Our data suggest that the combined treatment of BM-MSCs and hesperidin has a potent renoprotective efficacy against cisplatin-induced nephrotoxicity rather than the single treatment.

The kidney is the primary organ of the body performing vital functions to maintain hemostasis, regulate blood pressure, participate to detoxification and waste excretion. Therefore, the kidney is the main organ affected by toxic metabolites and excreted drugs, especially the chemotherapeutic agents resulting in kidney damage or nephrotoxicity (1).

Cisplatin or cis-diamminedichloroplatinum (II) is one the most effective chemotherapeutic drugs used to treat broad types of malignant tumors. However, cisplatin clinical use is greatly limited due to nephrotoxic complication (2). Cisplatin nephrotoxicity results from excessive reactive oxygen species (ROS) production, which induces oxidative stress leading to renal cytotoxicity, inflammation, and congestion (3). Toxic metabolites produced by cisplatin metabolism primarily damage renal tubular cells, resulting in DNA damage, mitochondrial dysfunction, and apoptosis. (4). The cisplatin-induced renal dysfunction is manifested by a significant increase of renal biochemical parameters such as urea and creatinine, and disturbance of electrolyte levels (5). 

Under certain conditions, bone marrow-derived mesenchymal stromal cells (BM-MSCs) can self-renew and differentiate into various functional cells. (6). The BM-MSCs' advantages of plasticity, ease of isolation from bone marrow, and multiplication into cell-derived colonies have opened a promising way of a novel therapeutic option of incurable and degenerative diseases (7). The therapeutic potential of BM-MSCs in acute renal failure is dependent on their ability to homing, engrafting, and differentiating the sites of damaged tissue. The homing occurs due to the interaction between the molecular signals released from the injured tissue as chemokines and receptors expressed on BM-MSCs (8). Thus, BM-MSCs accelerate the regeneration of the damaged tissue by promoting angiogenesis, stimulating mitosis, decreasing apoptosis, and reducing inflammation. They can also regulate the immune response and secrete potent paracrine factors that promote structural and functional renal recovery. BM-MSCs are considered as free-radical scavenging cells that can reduce oxidative stress by releasing exosomes to prevent ROS accumulation (9).

Hesperidin, a natural flavonoid antioxidant, is found in citrus and has antibacterial, anticancer, anti-inflammatory, and anti-apoptotic properties (10). Due to its antioxidant activity it can attenuate oxidative stress by inhibiting free radical formation (11). The hesperidin antioxidant activity was attributed to its hydrogen-donating properties, which neutralize hydroxyl and superoxide free radicals. As a result, much interest has been drawn to hesperidin as a renoprotective compound against cisplatin-induced renal damage (12). 

The current study aimed to assess the effects of BM-MSCs and hesperidin treatment alone or as a combination treatment to ameliorate cisplatin-induced nephrotoxicity and to restore renal structure and function in Wistar rats.

## Materials and methods

**BM-MSCs isolation, preparation, and culturing**

The BM-MSCs were isolated as previously described (13, 14). Briefly, male albino Wistar rats (4 weeks old, 60-80 g body weight) were used to isolate BM-MSCs. Rats were euthanized by an overdose of inhaled isoflurane anesthetic (15). After sterilizing the whole-body surface with 70% ethyl alcohol, the hind limbs (femur and tibia) were dissected and cleaned thoroughly from the adherent tissues by using sterile instruments. The bones were stored on iced Petri dishes containing Dulbecco's modified Eagle's medium (DMEM) (Life Science Production, UK) supplemented with 1% penicillin-streptomycin mixture (Lonza, Belgium). BM-MSCs isolation and culturing were done under proper sterile conditions in a vertical laminar airflow cabinet (Biobase, China). After cutting the bones just below the bone marrow cavity, the bone marrow cells were isolated by flushing with DMEM supplemented with 1% penicillin-streptomycin mixture. The bone marrow plug was then collected in a sterile 15 ml Falcon tube and centrifuged at 2000-3000 rpm for 3-5 min, and the step was repeated twice till obtaining a complete clear supernatant. The sediments were suspended in a complete DMEM medium supplemented with 15% fetal bovine serum (FBS) (Life Science Production, UK) (16). The viable cell counts were determined by staining them with 0.4% trypan blue solution and using a hemocytometer (17). The cellular suspensions were cultured in sterile 25 cm^2 ^cell culture flasks and incubated in a 5% CO_2_ humidified incubator (Biobase, China) at 37 ˚C. After 3-4 days incubation, the non-adherent cells were removed by replacing the old complete media with a sterile fresh one. 

On the 8^th ^day of incubation, the adherent cells (80-90% confluence) were washed twice by a sterile phosphate buffer saline (PBS) (Lonza, Belgium) to remove the excess FBS and trypsinized with 1.5 mL 0.25% trypsin/ 1 mM EDTA (Lonza, Belgium) at 37 ˚C for 2 min. The trypsin action was neutralized by adding 3-5 mL complete DMEM media to the suspension, followed by centrifugation and the clear supernatant was discarded. The cell pellet was re-suspended in 1 ml DMEM, and the cell viability was tested again.

**Morphological examination of BM-MSCs**

BM-MSCs were examined under the inverted microscope to identify their characteristic fusiform shape and well-developed cytoplasmic processes (18). 

**Gene expression analysis by real-time quanti-tative PCR (RT-qPCR)**

RNA was extracted from the cultured BM-MSCs using NucleoSpin RNA nucleic acids extraction kit (Macherey-Nagel, Germany). The cDNAs were synthesized from the mRNA by reverse transcription according to the manufa-cturer's instructions. Quantitative real-time PCR and data analysis were conducted using Step One real-time PCR Systromal (Applied Biosystromals, USA) and ViPrimePLUS One Step Taq RT-qPCR Green Master Mix I with ROX (SYBR Green Dye) (no. QLMM14-R) (Vivantis Technologies, Malaysia).

Ten μL of the master mix was prepared in 1x solution, and was added to 20 μL final volume containing 1 μL primers, 5 μL template cDNA, and 4 μL nuclease free water. Primers sequences specific for *CD105*, *CD73*, *CD34*, *CD45*, and the β-actin housekeeping gene were used (Table 1).

**Table 1:** Primers sequence for RT-qPCR

**Gene**		**Primer 5’ to 3’**	**Gene bank**
***CD105***	Forward	GGCAGCTTCAACAACCATCA	XM_032900290.1
	Reverse	GGATGGACTAGATCGGAGCC	
***CD73***	Forward	TGTTGGGACCAGCAACTCAA	NM_021576.2
	Reverse	TTTGAGGCTCAGTGGTAGCC	
***CD34***	Forward	GCGAGGCTTTCAACACAACC	XM_032903804.1
	Reverse	ACTCCACTGTCTTGATTCCC	
***CD45***	Forward	TGTGAACATACGGATTGTGA	AF251010.1
	Reverse	ACTTTAACTTAACAAACTGC	
**β-actin**	Forward	TGACAGGATGCAGAAGGAGA	NM_031144.3
	Reverse	TAGAGCCACCAATCCACACA	

The thermal cycling profile of RT-qPCR was set as follows: an initial cycle at 55 °C for 10 min, followed by a cycle for 8 min at 95 °C, then 40 cycles at 95 °C for 10 s and 60 °C for 60 s. The data were expressed as cycle threshold (Ct). The relative quantification of each target gene was quantified according to delta-delta Ct calculation (2^-ΔΔCt^) as following: 

ΔΔCt = [(Ct target, sample)- (Ct ref, sample)]- [(Ct target, control)- (Ct ref, control)] where: 

Ct target, control = Ct value of gene of interest in control DNA. 

Ct ref, control = Ct value of reference gene in control DNA.

Ct target, sample = Ct value of gene of interest in the tested sample.

Ct ref, sample = Ct value of reference gene in the tested sample.

**Animals**

Adult male albino Wistar rats (*Rattus norvegicus*) weighing 150-200 g were used. The rats were obtained from the Egyptian Company for the Production of sera and vaccines (Vacsera, Egypt). Animals were raised at the Faculty of Veterinary Medicine, Beni-Suef University, Egypt, in well-ventilated standard plastic cages, and maintained under standard laboratory conditions of controlled temperature (24±1 °C), humidity (50±5%), and 12:12 h light: dark cycle throughout the experiment. Food and water were provided *ad libitum*. Rats were kept for one week as an adaptation period before starting the experiment. Every effort was made to minimize the pain and animal suffering. All experimental procedures were conducted under the guidelines of the Institutional Animal Care and Use of Ethics Committee of Faculty of Veterinary Medicine, Beni-Suef University, Egypt (Ethical Approval Number: BSU/IACUC /2020/108)

**Experimental design**

Fifty healthy rats were divided into five equal groups (10 rats/group). Group-I served as control group and received carboxymethyl cellulose (CMC) (Sigma-Aldrich, USA) 1%,once daily via oral gavage for 4 weeks, and a single 1 ml distilled water intraperitoneally (IP) on the 5^th^ day of treatment (19), group-II received a single dose of cisplatin (7.5 mg/kg) IP to induce nephrotoxicity, group-III received a daily dose of hesperidin in CMC 1% (40 mg/kg) orally (20) for 4 weeks and on the 5^th ^day of administering hesperidin, cisplatin (7.5 mg/kg) was administered IP an hour before hesperidin administration. Group-IV (cisplatin-treated and BM-MSCs treated group) received distilled water orally once a day for 4 weeks, a single cisplatin dose (7.5 mg/kg) intravenously on the 5^th^ day, and then was injected with 1х10^6^ BM-MSCs cells/rat (BM-MSCs with ≥ 95% cell viability were used) once per week starting from the 6^th^ day till 4 weeks (13). Group V (cisplatin treated and hesperidin/BM-MSCs group) received hesperidin and cisplatin as group III and were concomitantly administered BM-MSCs as group IV the day after cisplatin injection.

At the end of the experiment (i.e. 4 weeks), venous blood samples were collected from rats under isoflurane anesthesia for measuring the biochemical parameters. Kidney tissues were obtained from euthanized rats for histological and immunohistochemical examination. 

**Determination of serum creatinine and urea levels**

The serum creatinine and urea levels were measured by the colorimetric method utilizing Diamond reagents Kits (Diamond Diagnostic Chemical Company, Egypt) (21). 

**Determination of serum sodium and potassium levels**

Serum sodium and potassium levels were determined by Spectrum-Diagnostics Sodium reagents (Spectrum Diagnostics, Egypt) using the colorimetric method (22). 

**Histological and histochemical examination**

Collected kidneys were dissected, washed with 

physiological saline solution, cut into small pieces, and fixed in 10% neutral buffered formalin. The fixed tissues were dehydrated in ascending alcohol grades, cleared in xylol, impregnated, and embedded in Paraplast®. Paraplast blocks were sectioned at 4‒5 µm thin layers, then stained with hematoxylin and eosin (H&E) to examine the general histological structure, periodic acid - schiff (PAS) for demonstration of the neutral mucopolysaccharides, and bromophenol blue to demonstrate the total protein content. Staining procedures were performed as outlined by Suvrna *et al*. (23). Kidney sections were examined and photographed using Leica binuclear research microscope (Leica, Hannover, Germany) attached with Canon digital camera (Canon, Japan).

The morphometric analysis was performed under light microscopy. For semiquantitative analysis of the severity of histopathological changes of the kidney, ten high-magnification (×400) fields of the cortex and medulla were randomly selected. Kidney damage was defined as glomerular atrophy, tubular epithelial vacuolar degeneration, desquamation, loss of brush border, hyaline cast formation, infiltration of inflammatory cells, and vascular congestion. Kidney histological changes were scored from (0-4) with the following semiquantitative scale: 0: no histological changes and the tissues appeared normal; 1: histological changes in less than 25% studied microscopic domains; 2: histological changes in 26-50% studied microscopic domains; 3: histological changes in 51- 75% studied microscopic domains; and 4: histological changes in more than 75% studied microscopic domains (24).

**Immunohistochemistry**

The p53 protein immunohistochemical staining was performed using the avidin-biotin-peroxidase method (25). Briefly, the tissue sections were deparaffinized and rehydrated using xylene and alcohol, respectively, and were then boiled in 10 mM sodium citrate (pH 6) for 8-15 min to retrieve antigens. The tissues were then immersed in 3% hydrogen peroxide for 15 min at room temperature to block endogenous peroxidase activity, followed by washing in double-distilled water and phosphate-buffered saline with 0.05% Tween® 20 (PBST). After that, the sections were incubated with a 1:20 dilution of p53 mouse monoclonal antibody (Invitrogen, Thermo Fisher Scientific, USA) for 1 h at room temperature. After intense washing by PBST, the slides were incubated with HRP-conjugated secondary antibody (Thermo Scientific, USA) and rinsed again with PBST. Finally, the 3.3-diaminobenzidine (DAB) solution (Dako, Denmark) was added, then the slides were rinsed with tap water and counterstained with Mayer's hematoxylin, dehydrated, and mounted. The negative control sections were processed similarly but without adding the primary antibody. The p53 protein expression in the immunostained sections was examined under a light microscope. The brown color immunostaining determined the positive reaction in the cytoplasm and nucleus.

**Quantification of histochemical and immun-ohistochemical staining**

Ten different areas of each PAS, bromophenol blue, and p53 stained images (X400)/ each group was analyzed by the Image J software (National Institute of Health, Bethesda, Maryland, USA), to estimate PAS, bromophenol blue color intensity, and p53 immunopositivity of the renal tissue in different experimental groups.

**Statistical analysis**

The obtained data were expressed as mean ±SEM (n=10), and all values were analyzed by SPSS Version 25 software package (SPSS, Inc., USA) using one-way ANOVA followed by Duncan's test post hoc analysis. P values <0.05 were considered as significant.

## Results

**Figure 1:**
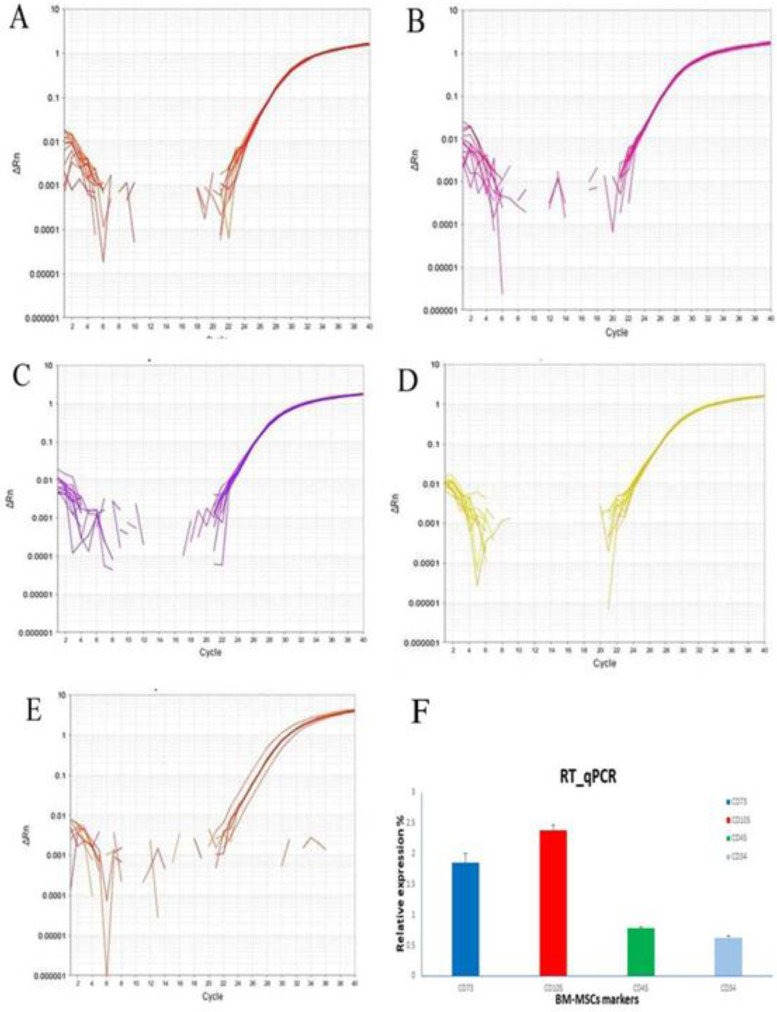
**Real-time quantitative PCR (RT-qPCR) amplification plot curves for** ***in vitro*** **expression profile of BM-MSCs markers.** A) *CD73* (red plot); B) *CD105 *(pink plot); C) *CD45 *(purple plot); D) *CD34 *(yellow plot); E) β-actin;F: gGene expression data analysis of BM-MSCs markers (*CD73*, *CD105*, *CD45*, and *CD34*) in relation to β-actin as a housekeeping gene. Data are expressed as mean ± standard error

RT*-*qPCR was performed to characterize the isolated BM-MSCs. BM-MSCs showed a high expression of specific markers of bone marrow derived stromal cells (CD73 and CD105) and weak expression of CD45 and CD34 (hematopoietic markers) (Figure 1F).

**Histological and histochemical investigations**

**Figure 2.**
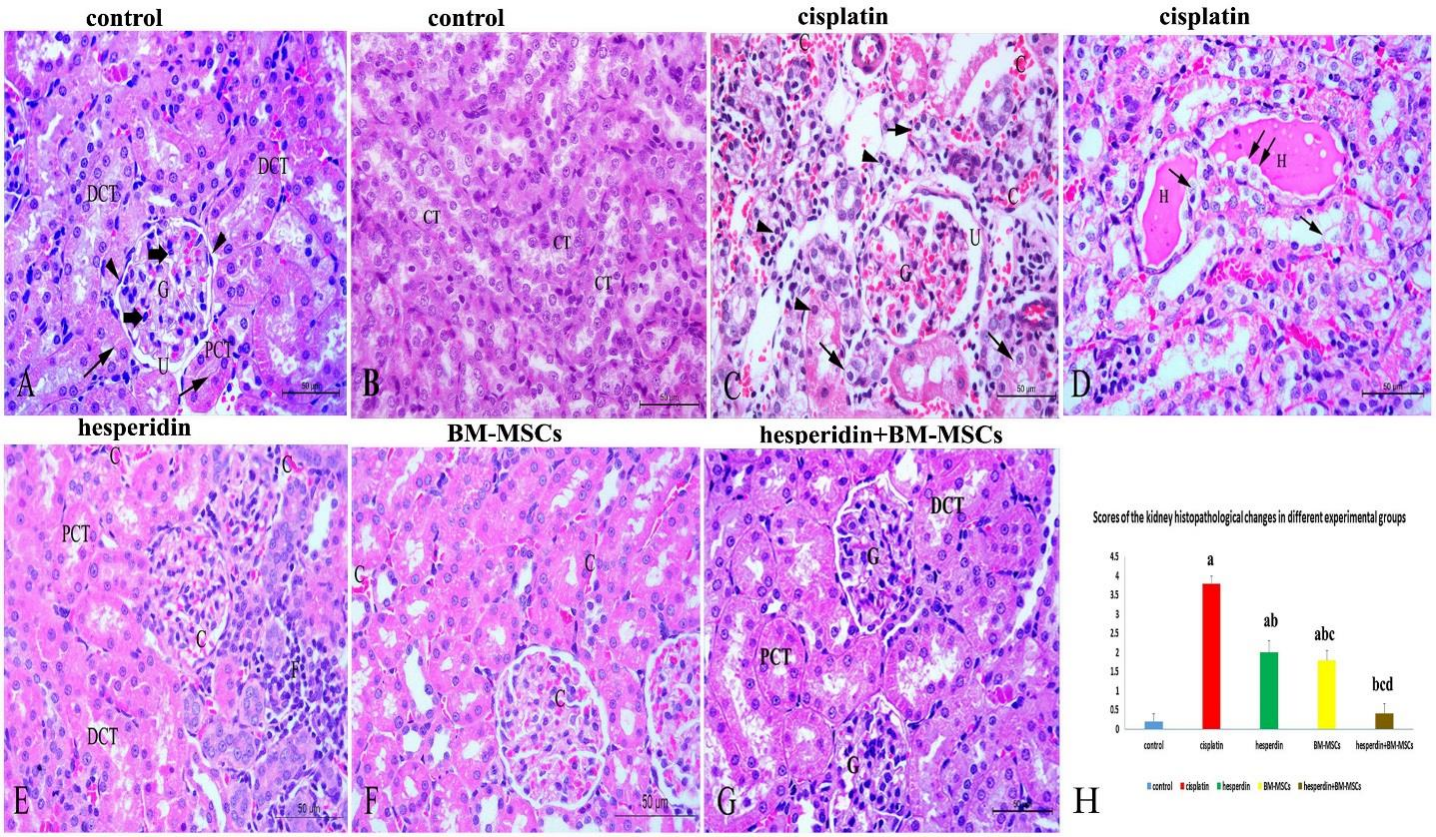
**Representative hematoxylin and eosin staining, and scores for renal injury.** A and B: Kidney sections of control rats; A) The renal cortex appeared with normal renal architecture including renal corpuscles with normal glomerulus (G), normal Bowman’s capsule having well-developed outer parietal layer lined with simple squamous epithelium (arrowheads), and inner visceral layer lined by podocytes and mesangial cells (thick arrows) separated by a urinary space (U). The renal tubules had the typical characteristics features of proximal convoluted tubules (PCT) and distal convoluted tubules (DCT) with prominent apical brush borders (thin arrows); B) The renal medulla, which was lined with simple cuboidal cells and had normal collecting tubules (CT), had rounded vesicular nuclei and acidophilic cytoplasm; C and D: kidneys of cisplatin-treated rats; C) The renal cortex showed severe degenerations, renal corpuscles with atrophied glomeruli (G) and a wide urinary space (U), cytoplasmic vacuolations and hydropic degenerations (arrows), nuclear pyknosis (arrowheads) and loss of the apical brush borders of the renal tubular epithelium, glomerulus and cortical blood vessel congestion (C), and diffuse lymphocytic infiltrations; : the renal medulla shows dilatations and cellular degenerations (arrows) of the renal tubules and their lumina filled with acidophilic hyaline materials (H); E: the kidneys of hesperidin administered rats show that most renal corpuscles have intact glomeruli and Bowman’s capsules and the epithelial cells of proximal tubules (PCT) and distal tubules (DCT) exhibit vesicular nuclei and few cytoplasmic vacuolations despite the presence of vascular congestion (C) and lymphocytic infiltration (F); F) The kidneys of BM-MSC-treated rats had improved renal tissue with a marked decrease in lymphocytic infiltrations. However, some congestion in glomerular tufts and blood vessels (C) was present; G: the kidneys of hesperidin and BM-MSCs-treated rats (combined treatment) showed complete regeneration of renal tissue that looked similar to the control group with normal renal corpuscles with normal glomerulus (G), proximal tubules (PCT), and distal tubules (DCT) with absence of inflammatory cells and congestion; H: the bar graph shows the scores of the pathological changes. Results are expressed as means ± SD with dissimilar superscript letters (significantly differing at P < 0.05); a: significantly different from the control group; b: significantly different from the cisplatin administered group; c: significantly different from hesperidin -treated group; d: significantly differ from BM-MSCs -treated group. Scale bar: 50 µm

The microscopic examination of the control rat kidney stained with H&E revealed normal renal corpuscles and tubules. The renal corpuscles had well definitive glomeruli (glomerular tufts) surrounded by a well-organized double layer of Bowman's capsule. The outer parietal layer was lined by simple squamous epithelium while podocytes and mesangial cells lined the inner visceral layer; the space between both layers is called the urinary space (Bowman's space). The renal tubules appeared with the typical proximal convoluted tubules pattern, distal convoluted tubules, and collecting ducts. The proximal convoluted tubules were small-sized in diameter, with narrow lumina, and lined by pyramidal cells with apical brush borders. The cytoplasm was deeply acidophilic, containing rounded vesicular nuclei. The distal convoluted tubules were large-sized in diameter, with wide lumina and lined by simple cuboidal epithelium with rounded central vesicular nuclei surrounded by lightly stained acidophilic cytoplasm. The loop of Henle's and collecting ducts of the renal medulla lined with simple squamous to cuboidal epithelium with flattened to rounded vesicular nuclei, respectively (Figure 2 A, B). 

However, the examined renal sections of the cisplatin-treated rats (group II) showed severe degenerative changes in the renal corpuscles and tubules. Most renal corpuscles appeared with atrophied glomeruli with prominent wide urinary space, nuclear pyknosis in podocytes and mesangial cells, irregularity, and partial loss of the parietal layer of Bowman's capsule. Moreover, pronounced loss of the renal tubule's cellular architecture was observed. The affected tubular epithelial cells exhibited swelling, cytoplasmic vacuolations, and hydropic degeneration with pyknotic nuclei in addition to partial and complete loss of the apical brush borders. Some renal tubules showed dilatation with acidophilic hyaline materials in their lumina. Also, there were intense pericorpuscular and peritubular lymphocytic infiltrations in addition to marked congestion in the glomerular tufts and all cortical blood vessels (Figure 2 C, D). 

In the hesperidin-treated rats (group III), most renal corpuscles appeared with intact glomeruli and Bowman's capsules. Nearly all tubular epithelial cells had vesicular nuclei and few cytoplasmic vacuolations. On the other hand, moderate interstitial infiltration of inflammatory cells and congestion in the glomerular tuft and renal blood vessels were present (Figure 2E). Interestingly, the BM-MSCs treated rats (group IV) showed a progressive renal tissue improvement in renal corpuscles and renal tubules. A marked decrease in the infiltrated inflammatory cells was also observed compared to that in the hesperidin-treated rats (group III); however, mild vascular congestion was still noticed (Figure 2F). 

The combined treatment with hesperidin and BM-MSCs (group V) showed a marked improvement of the renal tissue. It appeared similar to the control group with a pronounced absence of inflammatory cells and congestion. The renal tubules and corpuscles recovered their normal architecture with preserved brush borders of the tubular cells and the parietal layer of Bowman's capsule (Figure 2G).

**Figure 3:**
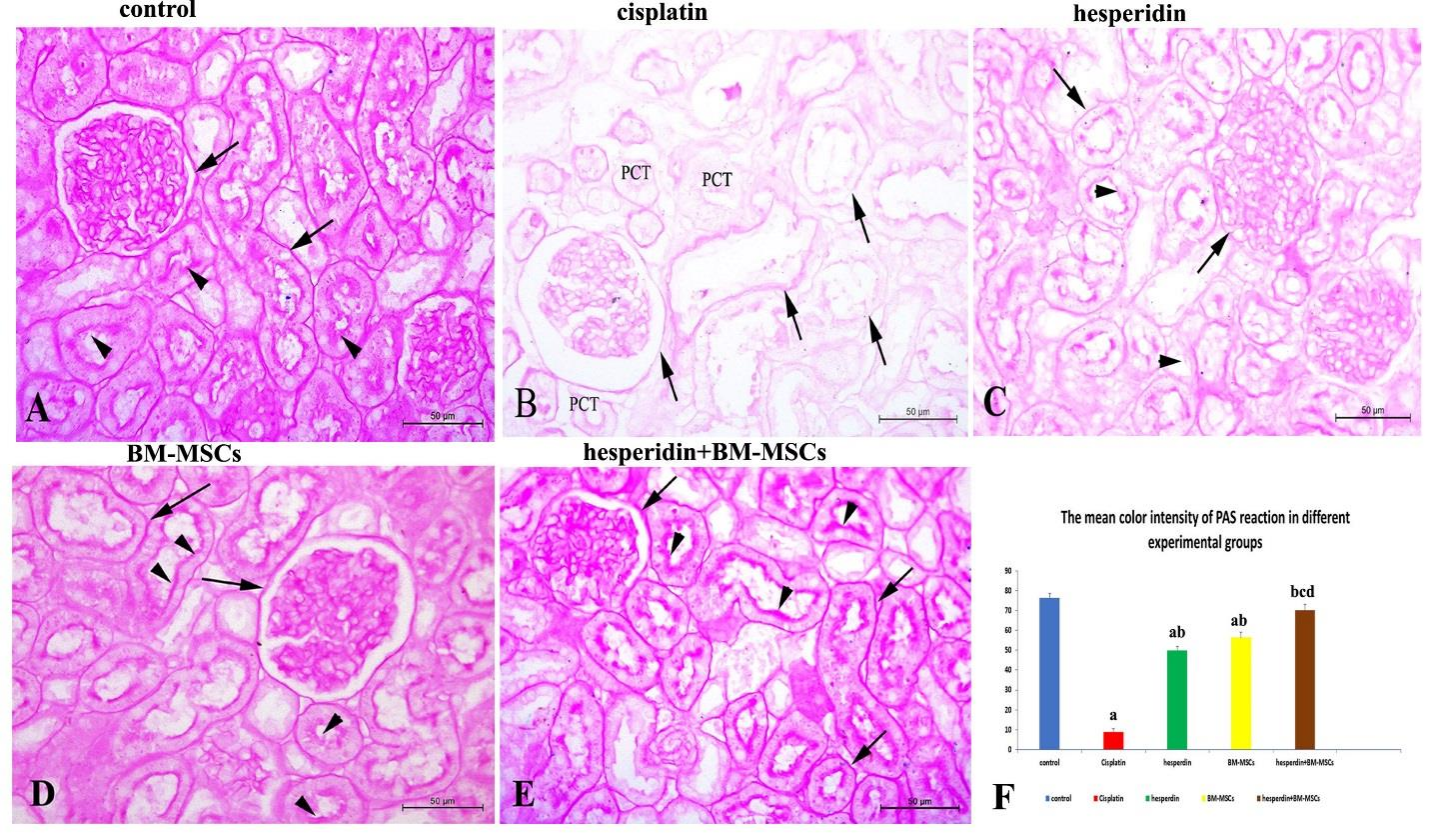
**Representative photomicrographs of renal tissues submitted to PAS staining for mucopolysaccharides evaluation**. A) Control rat kidney showed an intense PAS positive reaction (intense magenta) in the brush border (arrowheads) and the basement membrane of the renal tubules, as well as the basal lamina of the renal corpuscle in the control rat kidney (arrows); B) Cisplatin-treated rats showed a negative PAS reaction with loss of the brush border of the proximal tubules (PCT), while the basement membrane of the renal corpuscle and renal tubules were moderately reacting (arrows); C) Hesperidin-treated rats kidneys exhibited a mild to moderate PAS reaction of the brush border (arrowheads) and basement membrane of the renal tubules and the basal lamina of the renal corpuscle (arrows); D) BM-MSCs-treated rat's kidneys showed a moderate to strong PAS positive reaction in the brush border (arrowheads) and basement membrane of the renal tubules and renal corpuscles (arrows); E) Hesperidin and BM-MSCs treated rats kidneys having an intense PAS reaction similar to the control group in the brush border (arrowheads) and basement membrane of the renal tubules and the renal corpuscle (arrows); F) The bar graph shows the mean color intensity of PAS reaction in different experimental groups. Results were expressed as means ± SD with dissimilar superscript letters (significantly differing at P < 0.05); a: significantly different from the control group; b: significantly different from the cisplatin administered group; c: significantly different from hesperidin -treated group, d: significantly different from BM-MSCs -treated group. Scale bar: 50 µm

Figure 2H demonstrates the scoring results of the histopathological changes of the kidney in different experimental groups. There was a significant increase (P <0.05) in pathological changes in the cisplatin intoxicated group in comparison with the control and other treated groups. Renal pathological changes significantly decreased (P <0.05) in the BM-MSCs-administered group in comparison with the hesperidin-treated group. Moreover, substantial recovery of the renal architecture was observed in the group that received the combined treatment in comparison with those that were administered with hesperidin or BM-MSCs separately, and was non-significantly different from the control group. The PAS technique demonstrated mucopolysaccharides in the renal sections of the control group with a strong reaction to the PAS at the brush border, basement membrane of the renal tubules, and the basal lamina of renal corpuscle that appeared with dense magenta coloration (Figure 3A). In the cisplatin-treated rats (group II), the degenerated renal tubules appeared with partial or complete loss of the brush borders, which were negatively reacting with PAS. However, the renal corpuscles and tubule's basement membrane showed weak to moderate reaction (Figure 3B). 

Moderate improvement of the mucopoly-saccharides was observed in the renal sections of hesperidin-treated rats (group III). Some renal tubule's brush borders moderately reacted with PAS (Figure 3C). In the BM-MSCs treated rats (group IV), the renal tissue's mucopolysaccharides significantly improved in comparison with the hesperidin-treated rats (group III). The renal basement membranes and brush borders showed a marked increase in PAS reaction intensity from moderate to strong reaction (Figure 3D). The combined hesperidin and BM-MSCs treatment (group V) renal tissues reattained to a great extent the intense PAS-positive reaction, which was similar to the control group (Figure 3E) with preserved tubular brush borders and basement membranes of the renal corpuscles and tubules.

The mean color intensity of the PAS reaction in different experimental groups is represented in Figure 3F. A significant decrease (P <0.05) in the color intensity of the PAS reaction was noticed in the cisplatin-administered group in comparison with the control group and other treated ones. There was no significant difference between the hesperidin and BM-MSCs treated groups. Moreover, the PAS intensity reaction significantly increased (P <0.05) in the combined-treatment group in comparison with the groups that received hesperidin or BM-MSCs separately. A complete restoring of renal cytoplasmic carbohydrates occurred after applying the combined treatment with no significant difference in comparison with the control group.

**Figure 4:**
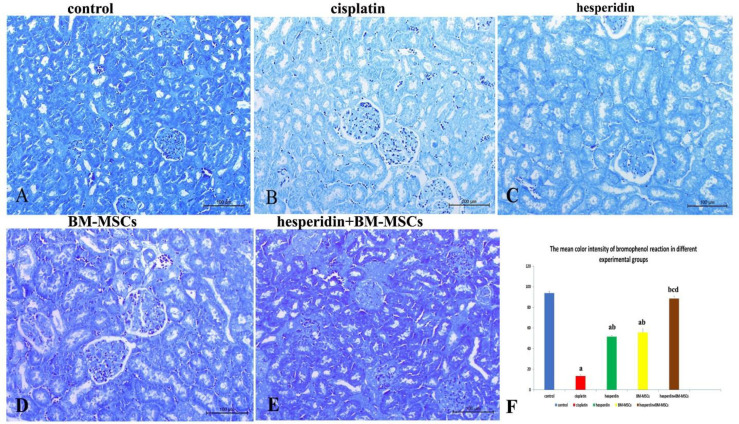
**Representative photomicrographs of renal tissues stained with bromophenol blue for total protein evaluation.** A) Control rat's kidneys have normal protein content, as evidenced by dense blue staining of renal corpuscles and renal tubules; B) Cisplatin-treated rat's kidneys have a marked decrease in protein content, as indicated by a faint blue reaction of the tubular cells and glomeruli; C) Hesperidin-treated rats kidneys have a moderate improvement in protein content, as evidenced by a moderate staining affinity of tubular cells; D) BM-MSCs-treated rats kidneys have a marked increase in protein content, as evidenced by moderate to strong blue staining of the renal tissue; E: hesperidin and BM-MSCs treated rats kidneys showed pronounced improvement in protein content with intense blue staining; F) The bar graph shows the mean color intensity of bromophenol blue in different experimental groups. Values are expressed as mean ± SD with dissimilar superscript letters (significantly differing at P < 0.05); a: significantly different from the control group; b: significantly different from the cisplatin administered group; c: significantly different from hesperidin-treated group; d: significantly different from BM-MSCs -treated group. Scale bar: 100 µm

The total protein content was screened using the bromophenol blue method. Kidney sections of control rats (group I) revealed normal total protein content in the cellular cytoplasm of renal tubules, mesangial cells and podocytes, which were stained deep blue (Figure 4A). The cisplatin-treated rats (group II) renal tissues showed a significant reduction of the total protein content in the cytoplasm of affected cells indicated by faint blue staining (Figure 4B). In contrast, kidneys in both single treatments with either hesperidin (group III) or BM-MSCs (group IV) showed moderate staining affinity of the cell lining renal tubules and corpuscles (Figure 4C, D). In the combined treatment with hesperidin and BM-MSCs (group V), the total protein content significantly improved in the renal corpuscles and tubular cells, as indicated by a strong staining affinity similar to the control group (Figure 4E).

Figure 4F demonstrates the morphometric analysis of the mean color intensity of the bromophenol blue reaction in different experimental groups. There was a significant decrease (P <0.05) in the color intensity of the bromophenol blue reaction in the cisplatin-administered group in comparison with the control group and other treated groups. No significant difference was present between the hesperidin, and BM-MSCs treated groups. Moreover, the bromophnol blue intensity reaction significantly increased (P <0.05) in the combined-treated group in comparison with the groups that received hesperidin or BM-MSCs separately. A complete restoring of the total proteins was occurred after the combined treatment.

**Immunohistochemistry of p53**

**Figure 5:**
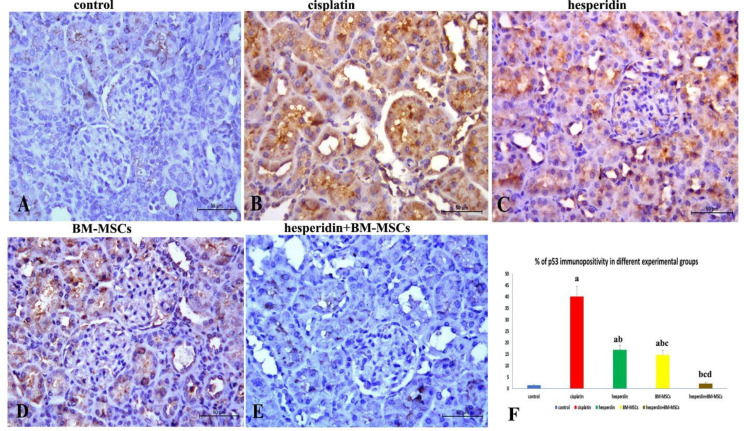
**p53 immunostaining** **of kidney sections.** A) Very few apoptotic (p53 positive) cells were present in the renal corpuscle cells and tubules in the control rat group, there are; B) cisplatin-treated rats exhibited very strong immunoreactivity with p53, which is manifested by a widespread dark brown color; C) hesperidin-treated rats exhibit moderate p53 immunoreactivity in the tubular cells; D) the BM-MSC-treated rat group exhibited weak immunoreactivity to p53; E: p53 immunostaining is barely detectable in the kidneys of hesperidin and BM-MSCs treated rat group; F: the bar graph shows the percentage of p53 immunopositivity in different experimental groups. Data are expressed as mean ± SD with dissimilar superscript letters (significantly differing at P < 0.05); a: significantly different from the control group; b: significantly different from the cisplatin administered group; c: significantly different from hesperidin-treated group; d: significantly different from BM-MSCs-treated group. Scale bar: 50 µm

The p53 protein immunohistochemistry of renal tissues showed that most renal corpuscular and tubular epithelial cells of the control group (group I) exhibited a weak reaction for the p53 (Figure 5A). However, in response to nephrotoxicity induced by cisplatin treatment (group II), a progressive and robust reaction to the p53 was noticed, with a widespread brown color in the affected renal cells (Figure 5B). The immunostained sections of the hesperidin-treated rats (group III) showed moderate reaction (Figure 5C), while the BM-MSCs treated rats (group IV) renal tissues reacted weakly in comparison with the cisplatin group (Figure 5D). The combined treatment with hesperidin and BM-MSCs (group V) showed a marked decrease in the renal apoptotic cells. The renal corpuscles and tubular cells reacted weakly to the p53 antibody (Figure 5E). Figure 5F shows the morphometric analysis of the percentage of p53 immunopositive apoptotic cells in different experimental groups. The rate of immunopositive p53 apoptotic cells is significantly higher (P <0.05) in the cisplatin-administered group in comparison with the control and other treated groups. A significant decrease in immunopositive apoptotic cells (P <0.05) in the BM-MSCs-treated group was observed in comparison with the hesperidin-administered group. Moreover, the immunopositive apoptotic cells significantly decreased (P <0.05) in the combined treated group in comparison with the single treated ones, while no significant difference with the control group was observed.

**Evaluation of biochemical parameters related to renal functions**

**Figure 6:**
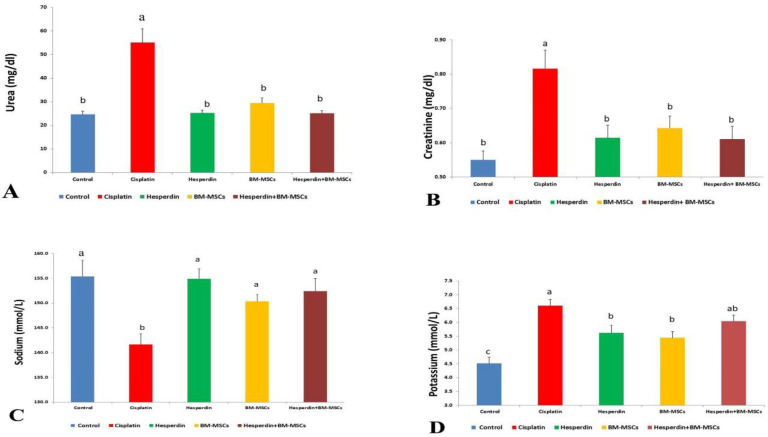
**Figure 6. The effect of BM-MSCs and/or hesperidin on serum biochemical parameters after cisplatin-induced nephrotoxicity. **A) Urea; B) Creatinine levels; C) sodium levels; D) Potassium levels. The data are presented as mean ± standard error (N=6). The mean values with different superscript symbols differ significantly (P <0.05).

Figure 6 demonstrates the effect of BM-MSCs and hesperidin treatments on the renal function parameters in cisplatin-treated rats. There was a significant increase in serum urea, creatinine, and potassium levels in the cisplatin-treated group (P <0.05) in comparison with the control group. In contrast, treatment of cisplatin-induced nephroto-xicity with hesperidin, BM-MSCs, and their combination for four weeks significantly reduced the serum urea and creatinine levels (P < 0.05) in comparison with the cisplatin-treated group (Figure 6A, B, and D). The serum sodium level exhibited a significant decrease in the cisplatin-treated group (P < 0.05) in comparison with the control group. However, a significant elevation of the sodium levels was noticed (P < 0.05) in the hesperidin,

BM-MSCs, and their combination treatment (Figure 6C)

## Discussion

The current study revealed that hesperidin, BM-MSCs, or hesperidin and BM- MSCs admini-stration significantly reduced the nephrotoxic effect of cisplatin. Moreover, co-administration of hesperidin and BM-MSCs had the most potent ameliorative impact on restoring the damaged renal tissue. 

The present study showed that cisplatin intoxication induced renal dysfunction as indicated by significant altered renal biochemical parameters, including blood urea, creatinine, potassium, and sodium levels (5). The biochemical findings were supported by the severe alteration of the renal architecture induced by cisplatin injection. Severely degenerated tubular epithelial cells, dilated renal tubules with intraluminal cast formation, atrophied glomeruli with wide urinary spaces, interstitial lymphocytic infiltrations, and congestion of glomerular renal blood vessels were observed (26). Additionally, significant depletion of the cytoplasmic mucopolysaccharides and protein content was confirmed by a weak PAS reaction and faint bromophenol blue staining **(**27). Our study confirmed the apparent apoptotic effect caused by cisplatin administration in kidney as detected by the strong immunopositivity reaction for p53 (4). Some studies reported that the tubular cell apoptosis and renal toxicity caused by cisplatin are due to the massive production of proinflammatory cytokines, including TNF-α (28). The apoptotic effect was more intense in the renal tubules, mainly proximal convoluted tubules, because of the high concentration of mitochondria and their functional role in concentration and reabsorption processes (27). Therefore, the renal tubules are the primary target for cisplatin-induced oxidative stress and activate apoptosis pathways (29). The massive production of ROS, free radicals, and inflammatory cytokine increase of oxidative stress act directly on the renal tubular cell components leading to cytotoxicity, tubular mitochondria damage, tubular transport system disturbances, protein synthesis inhibition, and DNA injury (28).

The imbalance between oxidant-antioxidant statuses caused by cisplatin has led several studies to recommend providing exogenous natural antioxidants to enhance the renal antioxidant defense system, and protect kidneys from nephrotoxicity **(**30,31). Several studies investigated the administration of exogenous antioxidants, especially the natural type, to enforce the renal antioxidant defense and avoid nephrotoxicity induced by nephrotoxic drugs (32). It is generally accepted that natural antioxidants significantly improve or prevent nephrotoxicity by trapping destructive free radicals and inhibiting inflammation (31).

In the current study, the treatment of cisplatin-intoxicated rats with hesperidin antioxidant substantially improved the renal biochemical parameters in comparison with the cisplatin- treated group similar to the findings of Kumar et al. (30). Regarding the histopathological findings, the degenerative changes in renal corpuscles and tubules decreased while cytoplasmic mucopolysaccharides and protein content were moderately restored. Additionally, vascular congestion and lymphocytic infiltrations persisted. Our results coincide with previous studies that demonstrated that the antioxidant therapy using flavonoids, including hesperidin, Vitamin C, or selenium had a partial inhibitory effect on the cisplatin-induced oxidative stress in the kidney (33). The antioxidant effect of hesperidin is achieved by decreasing the ROS reaction by scavenging free radicals and chelating metals and converting them into non-toxic end products (12). Our study suggested that hesperidin showed nephroprotective capacities because of its antioxidant properties.

The use of BM-MSCs is a promising therapeutic strategy for repairing renal damage and restoring kidney function and structure (34). The BM-MSCs used in this work were characterized before being used in the experimental study. The prepared stem cells were spindle shaped with few and short cytoplasmic processes as previously described (14). Their marker expression profiles were confirmed by quantitative real-time PCR analysis of *CD105*, *CD73*, *CD34*, and *CD45*. The cultured BM-MSCs highly expressed *CD73* and *CD105 *(mesenchymal stromal cells) and lowly expressed *CD34* and *CD45* (hematopoietic) marker genes (35), confirming their biological chara-cteristics.

The BM-MSCs treatment alone after cisplatin administration (group IV) improved the renal biochemical parameters but with no significant difference was observed in comparison with the group that received hesperidin only. In contrast, the renal architecture was significantly retrieved in comparison with rats that received hesperidin only. BM-MSCs treated rats showed marked disappearance of inflammatory cells, decreased vascular congestion, reduced cellular apoptosis (i.e., less expression of p53). Our data supported the previous studies that showed a pivotal role of the BM-MSCs in kidney repair due to their ability to home the injured kidney, secrete anti-inflammatory cytokines, and their easy differentiation into functional tubular epithelial cells (36). Furthermore, the BM-MSCs showed the ability to prevent the pathological process of cisplatin-induced nephrotoxicity early, and reduce lipid peroxidation, which enhances the kidneys’ histological and functional regeneration (37).

New strategies are directed to enhance the treatment efficacy of BM-MSCs (38), and to overcome the low survival ratio of the transplanted stromal cells induced by their insufficient resistance against the oxidative and inflammatory stresses at the injured sites (31). Due to their insufficient resistance to oxidative and inflammatory stresses at the damaged sites, the transplanted stromal cells have low survival ratios, which is the crucial problem affecting stromal cell therapy (31). Hence, our results further confirm that the antioxidant pre-treatments is able to significantly increase the stromal cell longevity, viability, and repair efficacy (6). Studies showed that the treatment of diabetic rats with hesperidin and BM-MSCs together was the most potent approach in ameliorating deteriorated lipid profile, heart and kidney functions (13). Also, antioxidant preconditioning could effectively improve the therapeutic effect of adipose-derived mesenchymal stem cell therapy for liver fibrosis (39).

Interestingly, the current study showed that the pre-treatment of cisplatin-treated rats with hesperidin antioxidant followed by BM-MSCs injection remarkably improved the kidney function and renal architecture. All treatments, either separately or in combination, improved the renal function parameters after cisplatin administration with no significant differences. However, according to our histological assessment, we noticed a substantial restoration of the renal architecture compared to the single treatment, which became similar to the control group (40). 

Therefore, the current study revealed that combined treatment of hesperidin and BM-MSCs has the most significant restoring effect against the cisplatin nephrotoxicity than using them separately. 

In conclusion, the cisplatin-induced nephrotoxicity and biochemical renal parameters imbalance was significantly improved by the treatment with BM-MSCs, hesperidin, and their combination. Based on the histological, histochemical and immunohistochemical results, the combination of BM-MSCs and hesperidin was the most effective approach in curing cisplatin-induced nephrotoxicity in rats. The pre-treatment of hesperidin antioxidant significantly improved the BM-MSCs therapy for cisplatin- induced nephrotoxicity, suggesting this approach to enhance the stromal cell therapeutic efficacy.
